# Rectovaginal fistula in a cervical cancer patient treated with sequential radiotherapy and bevacizumab: A dose‐volume analysis

**DOI:** 10.1002/ccr3.3955

**Published:** 2021-02-23

**Authors:** Kento Tomizawa, Ken Ando, Hirofumi Shimada, Takuya Kaminuma, Kazutoshi Murata, Takahiro Oike, Tatsuya Ohno

**Affiliations:** ^1^ Department of Radiation Oncology Gunma University Graduate School of Medicine Gunma Japan; ^2^ Gunma University Heavy Ion Medical Center Gunma Japan; ^3^ QST Hospital National Institutes for Quantum and Radiological Science and Technology Chiba Japan

**Keywords:** bevacizumab, cervical cancer, dose‐volume analysis, radiation, rectovaginal fistula

## Abstract

Bevacizumab is a monoclonal antibody against vascular endothelial growth factor that exerts antitumor effect by preventing tumor angiogenesis. Gastrointestinal fistula is a common side effect of bevacizumab in combination with radiotherapy. This case of rectovaginal fistula indicates that the side effect may be unpredictable by the conventional dose‐volume parameters for the rectum.

## Graphical abstract

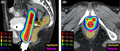

## 1 INTRODUCTION

We report a case of rectovaginal fistula after chemoradiotherapy for primary cervical cancer, followed by bevacizumab to treat metastatic tumors. Dose‐volume analysis suggested that radiation exposure in the fistulated region was unexpectedly low compared with the previously recognized tolerance dose for the rectum, raising awareness of bevacizumab use following radiotherapy.

Cervical cancer is the fourth most common cancer in women worldwide.^1^ Chemoradiotherapy is the standard definitive treatment for locally advanced cervical cancers.^2^, ^3^, ^4^, ^5^ On the other hand, addition of bevacizumab to the chemotherapy regimen (either cisplatin plus paclitaxel or topotecan plus paclitaxel) improves survival of those with recurrent or metastatic cancer.^6^ The downside is that bevacizumab increases the risk of gastrointestinal fistula (by approximately 7% for Grade‐2 or Grade‐3 fistulas based on the Common Terminology Criteria for Adverse Events [CTCAE] version 3.0) in patients with a previous history of pelvic irradiation.^6^ Therefore, practitioners should be aware of gastrointestinal fistula in cervical cancer patients treated with sequential radiotherapy plus bevacizumab. However, evidence pertaining to dose‐volume data for the rectum in this population is lacking. In fact, no study has mentioned whether the fistulated part of the rectum was corresponding to the dose hotspots. Here, we report a patient with cervical cancer who developed gastrointestinal fistula after treatment with definitive chemoradiotherapy followed by bevacizumab. We present a dose‐volume analysis for the rectum.

## 2 CASE REPORT

**FIGURE 1:**
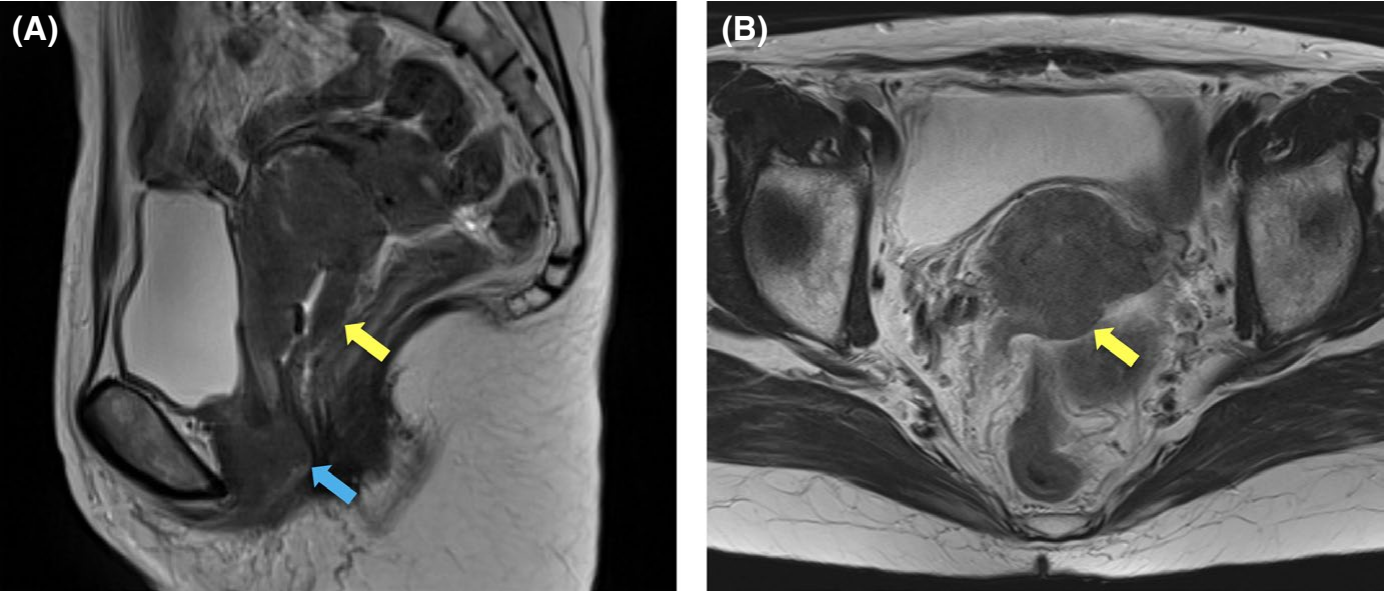
T2‐weighted magnetic resonance images of the primary cervical tumor taken at the time of diagnosis. (A) Sagittal plane. (B) Axial plane. Yellow arrows indicate the primary tumor at the cervix (57 × 48 × 69 mm). A blue arrow indicates the skip lesion in the anterior wall of the lower vagina (39 × 29 × 29 mm)

A 58‐year‐old Japanese woman, newly diagnosed with squamous cell carcinoma of the uterine cervix, was referred to the department of radiation oncology from the department of gynecology for definitive chemoradiotherapy. She had a history of rheumatoid arthritis (RA), which was treated with methotrexate and corticosteroid for 16 years. Bimanual pelvic examination revealed a tumor in the uterine cervix, with bilateral parametrial involvement not reaching the pelvic wall. In addition, a skip lesion was observed in the anterior wall of the lower vagina. T2‐weighted magnetic resonance imaging revealed a tumor in the uterine cervix (57 × 48 × 69 mm) and the skip lesion (39 × 29 × 29 mm) (Figure 1). ^18^F‐fluorodeoxyglucose positron emission tomography (FDG‐PET) combined with computed tomography (CT) showed increased FDG uptake by the tumor (maximum standardized uptake value [SUVmax], 11.7) and by the skip lesion in the lower vaginal wall (SUVmax, 7.0). There were no radiological findings indicative of lymph node or distant organ metastasis. Cystoscopy and colonoscopy revealed no tumor invasion of the bladder or rectum, respectively. Blood examination showed elevated levels of squamous cell carcinoma antigen (28.0 ng/ml), cytokeratin 19 fragment (5.1 ng/ml), and carcinoembryonic antigen (18.1 ng/ml). From these findings, the patient was diagnosed with stage IIIA cancer based on International Federation of Gynecology and Obstetrics (FIGO, 2009 version) criteria.

**FIGURE 2:**
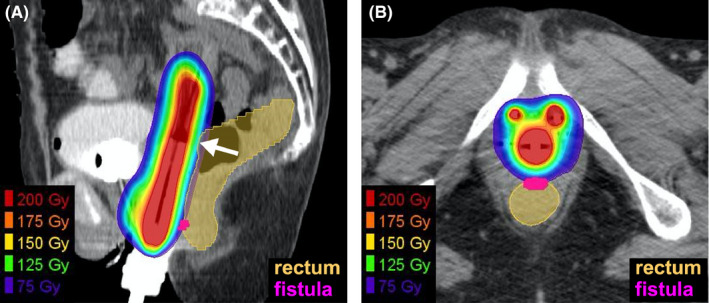
Distribution of the total equivalent dose in 2 Gy‐fractions, with the α/β ratio of 3 (EQD2_3_) from external beam radiotherapy (EBRT) and brachytherapy superimposed on the treatment planning computed tomography images for brachytherapy (third session). (A) Sagittal plane. (B) Axial plane. The arrow indicates the part of the rectum in which the total EQD2_3_ exceeded 75 Gy. EBRT plan was created using Eclipse (Varian, Palo Alto, CA, USA). Rigid registration of EBRT‐ and brachytherapy‐plans and calculation of the total EQD2_3_ were done using MIM Maestro version 6.9.3 (MIM Software Inc, Beachwood, OH, USA)

The patient received definitive concurrent chemoradiotherapy with cisplatin.^7^ Radiotherapy comprised external beam radiotherapy (EBRT) and brachytherapy. For EBRT, a total of 50.4 Gy was delivered in 28 fractions, five fractions per week; the latter 10.8 Gy was delivered using a 3 cm‐width central shield. The irradiation field for the EBRT included the primary tumor and common iliac‐, internal iliac‐, external iliac‐, obturator‐, and inguinal‐lymph node regions. Brachytherapy was performed using a high‐dose rate ^192^Ir remote‐after‐loading system (microSelectron, Elekta, Stockholm, Sweden) and in‐room CT‐based three‐dimensional treatment planning systems (Oncentra, Elekta, Stockholm, Sweden).^3^ A vaginal cylinder and two interstitial needles, targeting the skip lesion in the lower vaginal wall, were used. Four sessions were performed weekly. The first three sessions targeted the high‐risk clinical target volume (HR‐CTV),^8^ whereas the fourth session targeted the gross tumor to reduce the dose irradiated to the rectum. The D_90_ value (ie, the minimum dose at which any 90% of the volume is irradiated) of the target volume exceeded 6 Gy in each of the four sessions. For the rectum, the D_2cc_ value (ie, the maximum dose at which any 2 cc of the volume is irradiated) was below 6 Gy in each of the four sessions, for which the total equivalent dose in 2 Gy‐fractions with an α/β ratio of 3 (EQD2_3_) from EBRT plus brachytherapy^9^ was 75.4 Gy (Figure 2 and Table 1). Cisplatin (40 mg/m^2^) was administered weekly for a total of five courses. Radiotherapy was completed as planned. The treatment duration was 52 days. The following acute adverse events, based on the CTCAE version 4.0, were observed: a decrease in the white blood cell count (Grade 4); a decrease in the neutrophil count (Grade 3); radiation‐induced dermatitis (Grade 2); diarrhea (Grade 1); and oral mucositis (Grade 1). The cisplatin dose for the fifth course was reduced to 30 mg/m^2^ due to Grade 4 hematotoxicity.

**TABLE 1:** Summary of radiation dose delivered to the rectum

D2cc	EBRT	Brachytherapy				
		#1	#2	#3	#4	Total
Rectum	39.6 Gy/22 fr	5.7 Gy	5.8 Gy	5.9 Gy	4.5 Gy	75.4 Gy(EQD2)
Fistulated rectum	39.6 Gy/22 fr	4.2 Gy	4.4 Gy	4.6 Gy	1.4 Gy	57.7 Gy(EQD2)

Abbreviations: D2cc, the maximum dose at which any 2 cc of the volume is irradiated; EBRT, external beam radiotherapy; EQD2, equivalent dose in 2 Gy‐fractions with alpha/beta ratio of 3; fr, fraction(s).

**FIGURE 3:**
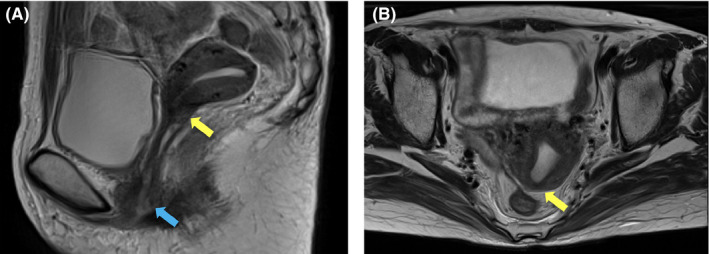
T2‐weighted magnetic resonance images of the primary cervical tumor taken at 3 months from initiation of radiotherapy. (A) Sagittal plane. (B) Axial plane. Yellow arrows indicate the site of primary tumor at the cervix observed in Figure 1. Blue arrow indicates the site of the skip lesion observed in Figure 1

**FIGURE 4:**
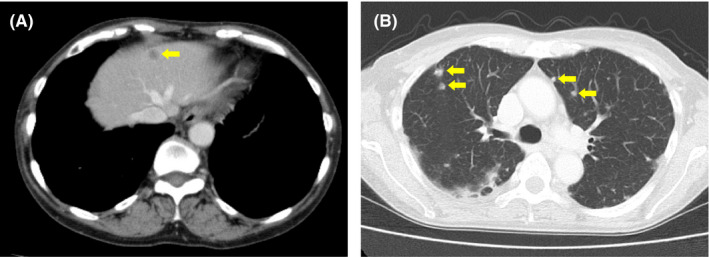
Computed tomography images taken at 5 months from initiation of radiotherapy. The arrows indicate metastatic tumors in the liver (A) and lung (B)

At 3 months after the start of treatment, a follow‐up CT showed almost complete remission of the primary tumor (Figure 3). However, a CT from the neck through the pelvis taken at 5 months showed multiple metastatic tumors in the liver and lung (Figure 4); no other metastases were identified. The patient was treated with cisplatin (50 mg/m^2^), paclitaxel (175 mg/m^2^), and bevacizumab (15mg/kg) for a total of two courses with an interval of 1 month.

**FIGURE 5:**
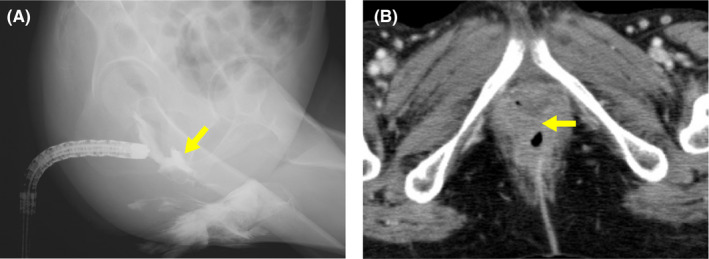
Radiological findings suggestive of rectovaginal fistula observed at 9 months from initiation of radiotherapy. (A) Barium enema radiography. The arrow indicates leakage of contrast enhancement agents (injected into the rectum) into the vagina. (B) Computed tomography. The arrow indicates the rectovaginal fistula

At 9 months, the patient experienced leakage of stool from the vagina. A barium enema and CT revealed a rectovaginal fistula (Figure 5). The patient underwent colostomy.

A CT taken at 10 months showed progression of the metastatic liver tumors, although the primary tumor remained controlled. The patient chose to receive best supportive care. At 1 year and 2 months, the patient died of primary disease.

We retrospectively delineated the fistulated part of the rectum and performed a dose‐volume analysis. The results showed that the D_2cc_ value for the fistulated part of the rectum was 4.2, 4.4, 4.6, and 1.4 Gy at each of the four brachytherapy sessions, for which the total EQD2_3_ was 57.7 Gy (Table 1).

## 3 DISCUSSION

Bevacizumab is a monoclonal antibody against vascular endothelial growth factor that exerts antitumor effect by preventing tumor angiogenesis. Bevacizumab has been shown to improve the outcome of the patients with gynecologic malignancies. Tewari et al reported that addition of bevacizumab to chemotherapy prolongs overall survival of the patients with metastatic, persistent, or recurrent cervical cancer (ie, 16.8 months versus 13.3 months, hazard ratio of 0.77).^6^ Tewari et al reported in a different study that addition of bevacizumab to chemotherapy prolongs overall survival of the patients with incompletely resected stage IV ovarian cancer (ie, 42.8 months versus 32.6 months, hazard ratio of 0.75).^10^ Evidence suggests that bevacizumab contributes to improve the outcome of the patients with other types of cancer; for example, Saltz et al reported that addition of bevacizumab to chemotherapy prolongs progression‐free survival of the patients with metastatic colorectal cancer (ie, 9.4 months versus 8.0 months, hazard ratio of 0.83).^11^ As such bevacizumab plays an important role in the treatment of various cancers.

A previous study by Strudza et al analyzed the association of dose‐volume parameters for the organs at risk with the incidence of gastrointestinal or genitourinary fistula caused by bevacizumab in the patients with cervical cancer who received definitive chemoradiotherapy as primary treatment, followed by chemotherapy with or without bevacizumab as secondary treatment for recurrent tumors.^12^ In that study, the incidence of gastrointestinal or genitourinary fistula was higher in bevacizumab group (40%, 4/10) than in no‐bevacizumab group (8%, 2/25). In the primary treatment, the total EQD2_3_ to D_2cc_ of the rectum or that to D_0.1cc_ was comparable between the two groups. However, that study did not mention whether the fistulated part of the rectum was corresponding to the dose hotspots. To pursue this issue, in our case, we performed a dose‐volume analysis of the fistulated part of the rectum. We found that the total EQD2_3_ to D_2cc_ for the fistulated part of the rectum was 57.7 Gy and that the fistulated part was not corresponding to the dose hotspot represented by the D_2cc_ of the whole rectum. This value of 57.7 Gy is unexpectedly low considering the data on dose‐volume constraints and the resultant rectal toxicity reported in previous studies (performed in the pre‐bevacizumab era), which employed the same radiotherapy strategy as used herein.^3^, ^13^ Ohno et al reported the outcome of definitive chemoradiotherapy for stage IB1–IVA cervical cancers (n = 80).^3^ In that study, the dose‐volume constraint for the rectum was set at a total EQD2_3_ to D_2cc_ of < 75 Gy, and no rectal toxicity greater than Grade 2 was observed after a median follow‐up of 60 months. Okazaki et al reported the outcome of definitive chemoradiotherapy for stage IB1–IVA cervical cancers (n = 103).^13^ In that study, the dose‐volume constraint for the rectum was set at a total EQD2_3_ to D_2cc_ of ≤ 70 Gy, and the 2‐year incidence of rectal toxicity greater than Grade 2 was 2%. Taken together, these data indicate that conventional dose‐volume constraints cannot be used to predict gastrointestinal fistula after sequential radiotherapy plus bevacizumab. Therefore, the risk for gastrointestinal fistula should always be recognized by practitioners who opt for sequential use of radiotherapy plus bevacizumab, regardless of the amount of radiation exposure to the gastrointestinal tract.

In our case, primary tumor at the uterine cervix had bilateral parametrial involvement not reaching the pelvic wall and a skip lesion in the anterior wall of the lower vagina. CT as well as MRI, and colonoscopy confirmed that the case was negative for rectovaginal septum involvement. The lesson to be learned from our case may be that unpredictable gastrointestinal perforation can occur in bevacizumab‐treated patients with cervical cancer with a history of definitive concurrent chemoradiotherapy even though rectovaginal septum involvement was negative. Unfortunately, the predictive factor of gastrointestinal perforation in such cases remains unelucidated at present, warranting further research.

The patient reported herein had RA, which was treated with methotrexate and corticosteroids. The effect of RA on adverse effects during radiotherapy is controversial.^14^, ^15^, ^16^ Lin et al analyzed radiation‐induced toxicity in 73 patients with collagen vascular disease (CVD), including 33 RA patients.^14^ In that study, the incidence of severe late toxicity was higher in RA patients than in background‐matched controls (29.7% *vs*. 13.9%, respectively). Chen et al analyzed the radiation‐induced toxicity in 36 CVD patients, including 17 RA patients.^15^ In that study, there was no significant difference in the incidence of late toxicity between RA patients and background‐matched controls. Ross et al analyzed radiation‐induced toxicity in 61 CVD patients, including 39 RA patients.^16^ In that study, there was no significant difference in the incidence of late toxicity between RA patients and background‐matched controls. Nevertheless, there is a possibility that the combination of RA, radiation, and bevacizumab contributed to the increased gastrointestinal vulnerability in our case.

## 4 CONCLUSIONS

Here, we report a case of rectovaginal fistula after concurrent chemoradiotherapy for the primary cervical cancer, followed by bevacizumab‐combined chemotherapy for the metastatic tumors. A detailed dose‐volume analysis suggested that the radiation exposure in the fistulated rectum was unexpectedly low compared with previously recognized tolerance doses for the rectum. This case indicates that practitioners should recognize the risk of gastrointestinal fistula during sequential treatment with radiotherapy plus bevacizumab, regardless of the amount of radiation exposure to the gastrointestinal tract.

## CONFLICT OF INTEREST

None.

## AUTHOR CONTRIBUTIONS

KT: analyzed clinical data, drafted the manuscript, and obtained funding; KA: analyzed clinical data; HS: analyzed clinical data; TK: treated the patient and analyzed clinical data; KM: treated the patient; T. Oike: supervised the study and finalized the manuscript; T. Ohno: supervised the study and obtained funding.

## ETHICAL APPROVAL

The enrollment of the patient was approved by the Institutional Ethical Review Committee of Gunma University Hospital (approval number: H2019‐226). The Institutional Ethical Review Committee waived the requirement for written informed consent from the patient because of the retrospective and noninvasive design of the study. The study was conducted in accordance with the ethical principles of the Declaration of Helsinki.

## Data Availability

The raw data presented in this study are not available to readers due to the restriction based on the approval by the Institutional Ethical Review Committee of Gunma University Hospital (approval number: H2019‐226).
